# Evaluation of Three Methods for the Treatment of Dentin Hypersensitivity: A Randomised Clinical Trial

**DOI:** 10.1016/j.identj.2024.03.013

**Published:** 2024-04-12

**Authors:** Narges Naghsh, Arezoo Hosseini, Azin Bazmara, Reza Birang

**Affiliations:** a Department of Periodontology, Dental Implants Research Center, Dental Research Institute, School of Dentistry, Isfahan University of Medical Sciences, Isfahan, Iran; b Dental Students’ Research Committee, School of Dentistry, Isfahan University of Medical Sciences, Isfahan, Iran

**Keywords:** Dentin hypersensitivity, Laser therapy, Diode laser

## Abstract

**Objectives:**

In this study, we aimed to compare the effectiveness of Gluma and high-power 980-nm diode laser, alone or in combination, in the treatment of cervical dentin hypersensitivity.

**Methods:**

A total of 20 patients (5 men and 15 women), aged 25 to 60 years, who met the inclusion criteria, were enrolled in this study. A total of 60 teeth were randomly divided into 4 groups: G1, 980 nm diode laser (in 2 sessions within a 1-week interval); G2, Gluma (in 2 sessions within a 1-week interval); G3, 980 nm diode laser plus Gluma; and G4: control. Thermal (cold spray) and air blast (air syringe of dental unit) stimuli were used to evaluate cervical dentin hypersensitivity in the patients. Their pain response was assessed using a visual analogue scale (VAS) before treatment (baseline), in the first treatment session (15 minutes after treatment), in the second treatment session (after 1 week), and in 2-week, 1-month, and 3-month follow-up sessions. The obtained data were analysed using non-parametric tests, including Kruskal–Wallis test, Friedman test, Mann–Whitney test, and Wilcoxon test, in SPSS Version 22 at a significance level of *P* < .05.

**Results:**

Based on the results, there was a significant difference in the average VAS scores for cold and air blast stimuli between the 4 groups 1 month after the intervention (*P* < .05). Meanwhile, the laser group had the lowest VAS score for cold and air stimuli. On the contrary, no significant difference was found between the 4 groups 3 months after the intervention (*P* ˃ .05).

**Conclusion:**

The present results showed that 980-nm diode laser alone was more effective than the other 2 intervention methods for 1 month.

**Trial registration:**

The study was registered in the Iranian Registry of Clinical Trials (IRCT20120901010703N5).

## Introduction

Cervical dentin hypersensitivity (CDH) is a common condition that causes a brief, sharp pain in response to various oral stimuli, including thermal changes (eg, cold temperatures), airflow, tactile stimuli, osmotic changes, and chemical exposure. CDH could occurs when dentin tubules become exposed due to enamel loss or cemental loss as a result of various factors comprising gingival recession, abrasion, attrition, abfraction, erosion, and improper brushing, and the pain cannot be explained as arising from any other form of dental defect or pathology.^1^ The prevalence of CDH has been reported to exceed 33.5% in the general population.^2^ The most widely accepted theory for explaining the mechanism of CDH is the hydrodynamic theory, which was proposed by Brannstrom in 1964. According to this theory, different oral stimuli lead to the outward or inward displacement of dentin fluids, which activates the pulp nerve endings and causes a short, sharp pain, known as CDH.^3^

Many treatment methods for CDH are suggested, including using mouthwash and toothpaste with desensitising chemical agents comprising potassium nitrate, aluminum ferric, oxalate, carbonate, and fluoride compounds. As well as, methods that apply Physical agents such as fluoride varnish, bonding agents, and restorative treatments like composite.^4^ The mechanism of action for most of the treatment methods is to disturbance of the neural response to stimulus, like potassium salts (potassium nitrate) or block dentin tubules, like fluoride and oxalates components, which prevents the fluid flow within the tubules and reduces pain.^5^ Nevertheless, in the application of chemical desensitising materials, a complete blockage of dentin tubules and long-term or permanent effects have not been observed and they can be readily eliminated through exposure to acid, friction, or saliva in oral environment.^6^, ^7^, ^8^, ^9^ Moreover, the application of restorative materials in CDH is commonly suggested only if there is a tooth structure loss.^10^

Sealing of dentin tubules may be possible with the use of glutaraldehyde compounds, such as Gluma Desensitizer, comprising 5.1% glutaraldehyde and 36.1% hydroxyethyl methacrylate (HEMA). Glutaraldehyde in the Gluma reacts with albumin in the fluid of dentin tubules and coagulates, causing albumin precipitation. These conditions trigger the polymerisation of HEMA, resulting in the formation of deep tags within the dentin tubules and causing their complete or partial blockage.^11^^,^^12^ So far, the use of Gluma has been evaluated in various studies, and its positive impact on the treatment of CDH has been reported.^13^, ^14^, ^15^, ^16^

In recent years, the use of lasers as a complementary treatment has expanded in dentistry. Diode lasers with wavelengths of 805 to 980 nm are used widely for soft tissue surgery, endodontic treatments and they also give excellent haemostasis, and are effective in gingival depigmentation.^17^, ^18^, ^19^ The advantage of using diode laser including their effectiveness, ease of application, greater versatility because of their impact size, and lower cost compared to other types of laser systems.^20^ Laser therapy was first used as a CDH treatment in 1985.^21^ The desensitising mechanism of high-power lasers is mostly related to their ability to occlude dentin tubules by melting and re-crystallising the dentin surface, as well as evaporating the tubule fluid, which subsequently disturbs the neural response.^22^

The advantages of combined methods have been also evaluated in some previous studies. In this regard, a study by Narayanan et al.^23^ found that combining 5% potassium nitrate with low-level laser therapy was more effective in reducing CDH than using either treatment alone. However, there is still no gold standard method, and there is a lack of adequate in vivo evidence for treatment of CDH. The results of previous studies are also heterogeneous, which indicates the need for further clinical trials to evaluate the effectiveness of different treatment methods.^22^ Therefore, in this study, we aimed to evaluate and compare the effects of a high-power 980-nm diode laser and Gluma, both alone and in combination, on patients with CDH in a 3-month follow-up. The null hypothesis in this study was there was no difference between the treatment groups in different times.

## Materials and methods

### Study design and ethical considerations

**Figure 1:**
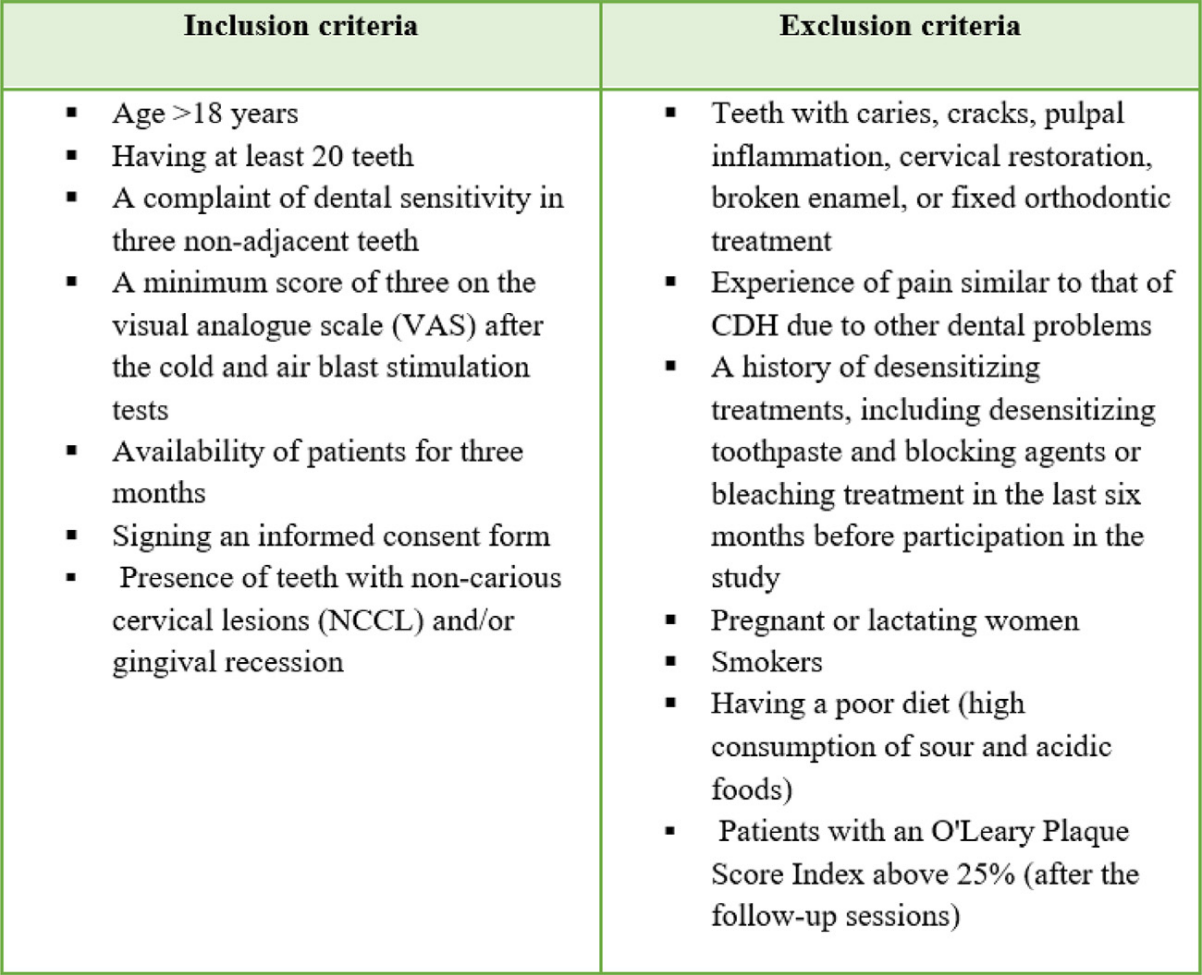
Inclusion and exclusion criteria.

This prospective, single-blind, randomised controlled clinical trial was conducted at the Department of Periodontics of the School of Dentistry, Isfahan University of Medical Sciences, from November 2022 until June 2023. The study was approved by the Research Ethics Committee of the university (IR.MUI.RESEARCH.REC.1400.384) and registered in the Iranian Registry of Clinical Trials (IRCT20120901010703N5). All the participants were informed about the study process, purpose, and the duration of the study, and signed informed consent forms in accordance with the Declaration of Helsinki.^24^ To confirm the diagnosis of CDH, individuals who reported dental sensitivity or pain were subjected to a clinical dental examination (by a single experienced evaluator [N.N]) by probing the teeth, assessing the response to cold and air blast stimulation tests, and reviewing preapical radiography results. The inclusion and exclusion criteria,^25^^,^^26^ were as shows in Figure 1.

### Evaluation of pain and CDH

To evaluate the patients’ perception of pain, we used a 10-point VAS scale. This scale consists of a horizontal line on which the patient indicates their level of pain. The scale ranges from zero (no pain) on the far left to 10 (worst unbearable pain) on the far right.^27^

Additionally, to evaluate CDH, first, the teeth were isolated from the adjacent teeth with 2 cotton rolls mesially and distally to prevent false positive results. An air blast test was then performed using an air syringe of dental unit. Air was directed onto the cervical one-third area of the tooth surface from a distance of 10 millimetres for 3 seconds at a pressure of 40 psi (±10 psi). Subsequently, the patient's pain score was determined based on VAS.^28^ After a 5-minute interval, the sensitivity of the teeth to cold stimulation was assessed by spraying a cotton swab with cold spray and placing it onto the cervical one-third region of the tooth for 3 seconds. Next, the patients’ pain scores were appraised on VAS.^29^

### Sample size, randomisation, and blinding

The sample size for this study was calculated using the G Power1 program, the data obtained from a pilot study,^30^ with an alpha level set at 0.05, an effect size (d = 1.7), and a power level set at 0.8. A total of 15 sensitive teeth per group showed to be necessary. Randomisation was performed by a dental student (A.B), using a table of random numbers. Each tooth was assigned a coded paper that was unknown to the participant, and each tooth was allocated to 1 of the 4 groups. In this single-blind trial, the participants were not aware of the type of treatment they received. For those receiving the Gluma treatment or the control group, we used the laser guide beam (with the laser turned off) to simulate the laser treatment.^30^^,^^31^

### Clinical protocol

A total of 20 patients, with 3 non-adjacent teeth (60 teeth) were randomly divided into the following 4 groups (15 teeth):

*High-power 980-nm diode laser group (n = 15)*: In this group, a diode laser (Fox Laser, A.R.C. Laser), with a wavelength of 980 nm and a power of 1 w, was used to irradiate the tooth surface from a distance of 1 mm for 30 seconds in a continuous mode (Energy was 30 J and the energy density for total area of tooth was 10.8 J/cm^2^). The cannula (fiber diameter:320 μm) was moved back and forth at a 45-degree angle during the procedure. Two sessions of treatment were conducted within a 1-week interval.^32^

*Gluma Desensitiser group (n = 15)*: First, the tooth requiring treatment was isolated using cotton rolls. It was then dried in oil-free and dry air for a duration of 3 seconds. Afterward, the desensitiser agent (Gluma Desensitizer) was gently rubbed on the tooth surface, especially on the cemento-enamel junction (CEJ), as well as the exposed cementum and dentin areas for 60 seconds. It was then dried in oil-free and dry air for 5 seconds until its shiny appearance disappeared. This process was repeated for 2 sessions within a 1-week interval.^33^

*High-power 980-nm laser + Gluma Desensitiser group (n = 15)*: For this group, the procedure used for the second group was repeated. After a 5-minute interval, the allocated tooth was irradiated under the same conditions as the first group.

*Control group (n = 15)*: No treatment was performed for this group. The laser guide beam (with the laser turned off) was used as a sham treatment.^31^

**Figure 2:**
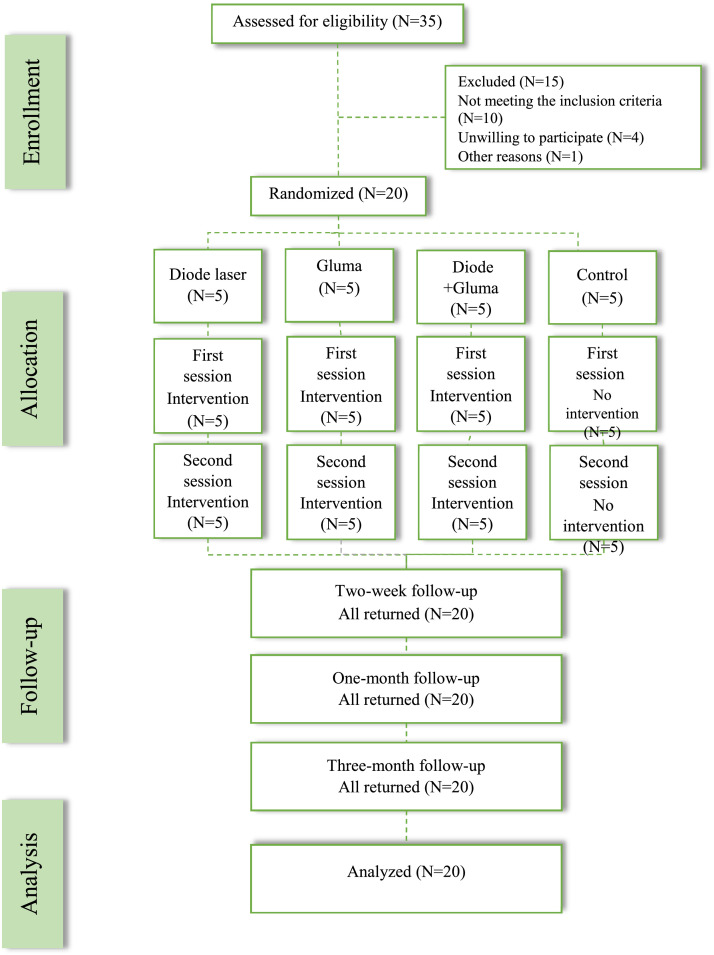
The study flowchart adapted from the CONSORT flowchart.

A single experienced evaluator (N.N). performed all the tests for CDH during the first visit at baseline (pretreatment), 15 minutes after the intervention (post treatment), during the second visit after the intervention (within a 1-week interval), and during 3 follow-up visits (after 2 weeks, 1 month, and 3 months, respectively) (Figure 2). All participants were advised to use a prescribed non-fluoridated toothpaste and to avoid brushing with excessive force and excess consumption of sour or acidic food.^31^^,^^33^

### Statistical analysis

All data were collected and analysed. The non-parametric Kruskal–Wallis test was used to analysed the data. For pairwise intra-group comparisons at different intervals, Mann–Whitney test was used. Moreover, Friedman test was performed to evaluate changes over time (from the pretreatment phase until 3 months after the intervention) within the 4 groups, and Wilcoxon test was used for pairwise comparisons between the groups at different intervals. Chi-square test was also used to compare the frequency distribution of qualitative data between the 4 groups, and 1-way analysis of variance (ANOVA) was conducted to compare the average age of the patients in the 4 groups. Data were analysed using SPSS Statistics 22.0, in all data analyses, a *P*-value < .05 was considered statistically significant.

## Results

All 20 participants (5 men and 15 women, aged 25-60 years) of this study completed the treatments and were followed-up for 3 months after the intervention. There were no reports of complications or discomfort during this time. The total VAS scores of CDH teeth for each group were determined in different intervals. The participants’ age and gender distributions are presented in Table 1. The results did not indicate any significant differences between the groups regarding these variables (*P* < .05).

**Table 1:** The participants’ characteristics based on age and sex.

Variables		Laser	Gluma	Laser + Gluma	Control	*P*-value
**Age (years)**		54.7 ± 60.54	37.1 ± 00.98	44.1 ± 00.75	43.13 ± 80.22	.121*
**Sex**	Female	4 (80%)	4 (80%)	3 (60%)	4 (80%)	.849†
	Male	1 (20%)	1 (20%)	2 (40%)	1 (20%)	

⁎ One-way analysis of variance (ANOVA).

† Chi-square tests were used to assess differences among groups.

The results of cold and air blast tests in the 4 groups, from pretreatment until 3 months after the intervention, are presented in Table 2. According to the results of Kruskal–Wallis test before the intervention (Baseline), there was no significant difference in mean VAS score in cold and air blast tests between the 4 groups (*P* = .917 and 0.809, respectively). Additionally, there was no significant difference between the 4 groups in the cold stimulation test 15 minutes and 1 week after the intervention or in the air blast test 15 minutes, 1 week, and 2 weeks after the intervention (*P* > .05). However, 2 weeks and 1 month after the intervention, the lowest mean VAS score for cold stimuli was observed in the laser group, while the highest mean VAS score was reported in the control group. Meanwhile, 3 months after the intervention, there was no significant difference between the 4 groups (*P* = .165). Also, based on the Friedman test results, within 3 months after the intervention, a significant reduction in cold sensitivity was found in each of the 4 groups (*P* < .05) (Table 3). For the air blast test, the average VAS score at different intervals showed a significant difference in the laser **+** Gluma group (where Friedman test was close to significance) 2 weeks and 1 month after the intervention compared to the baseline.

**Table 2:** The participants’ average VAS scores for cold stimulation and air blast tests in the groups.

Cold stimulation test						Air blast test				
	Laser	Gluma	Laser + Gluma	Control	*P*-value*	Laser	Gluma	Laser + Gluma	Control	*P*-value*
Baseline	5.27 ± 1.38	5.30 ± 1.63	5.20 ± 1.01	5.33 ± 2.02	.917	0.93 ± 0.70	1.53 ± 1.99	1.73 ± 1.67	1.27 ± 1.22	.809
15 min	4.40 ± 1.84	4.87 ± 1.96	4.67 ± 1.34	5.27 ± 2.12	.694	0.80 ± 0.56	1.33 ± 1.88	1.40 ± 1.29	1.73 ± 1.53	.242
1 W	3.73 ± 1.53	4.40 ± 1.80	4.27 ± 1.16	4.93 ± 2.15	.295	0.73 ± 0.46	1.27 ± 1.39	1.13 ± 0.99	1.47 ± 1.92	.746
2 W	2.93 ± 1.48	4.33 ± 1.80	3.87 ± 1.12	4.87 ± 1.13	.014	0.63 ± 0.50	1.07 ± 1.38	1.07 ± 0.96	1.67 ± 1.83	.239
1 M	2.80 ± 1.32	4.07 ± 1.53	3.73 ± 1.03	4.47 ± 1.99	.017	0.60 ± 0.51	1.13 ± 1.51	0.73 ± 0.70	1.80 ± 1.66	.025
3 M	3.33 ± 1.49	4.20 ± 1.57	3.87 ± 1.06	4.40 ± 2.03	.165	0.53 ± 0.51	1.07 ± 1.39	0.87 ± 0.35	1.53 ± 0.81	.213
*P*-value†	˂.001	˂.001	˂.001	˂.001		.297	.150	.05	.664	

min, minutes after the intervention; W, week after the intervention; M, month after the intervention.

⁎ From Kruskal–Wallis test.

† From Friedman test.

**Table 3:** Pairwise comparison of the average VAS scores in the cold stimulation test at different intervals in the 4 groups (*P* < .05).

**Laser**						
**Tim interval**	Baseline	15 min	1 W	2 W	1 M	3 M
Baseline		0.002	0.001	0.001	0.001	0.001
15 min			0.013	0.003	0.002	0.003
1 W				0.001	0.001	0.034
2 W					0.157	0.034
1 M						0.005
3 M						
**Gluma**						
Baseline		0.035	0.001	0.001	0.001	0.003
15 min			0.083	0.033	0.006	0.047
1 W				0.655	0.059	0.386
2 W					0.102	0.623
1 M						0.527
3 M						
**Laser + Gluma**						
Baseline		0.011	0.001	0.001	0.001	0.001
15 min			0.014	0.003	0.002	0.003
1 W				0.014	0.011	0.014
2 W					0.157	1
1 M						0.317
3 M						
**Control**						
Baseline		0.655	0.083	0.035	0.005	0.002
15 min			0.025	0.014	0.001	0.001
1 W				0.317	0.008	0.021
2 W					0.014	0.035
1 M						0.655
3 M						

**Chart 1:**
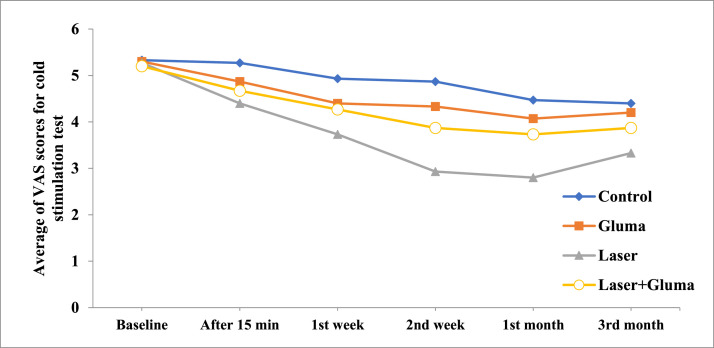
The participants’ average VAS scores for cold stimulation test in the groups.

**Chart 2:**
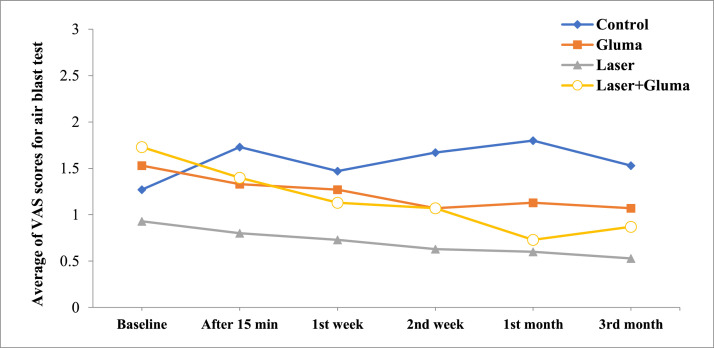
The participants’ average VAS scores for air blast test in the groups.

The results of Mann–Whitney test (for groups where the Kruskal–Wallis test was significant) showed that 2 weeks and 1 month after the intervention, the average VAS score for cold stimuli was significantly lower in the laser group compared to the control and Gluma groups and, 1 month after the intervention, the average VAS score for the air blast test was significantly higher in the control group compared to the laser, Gluma, and laser**+**Gluma groups respectively (Table 4). Chart 1, Chart 2 representing VAS scores of the study groups at different intervals.

**Table 4:** Intra-group pairwise comparison of the average VAS scores for the cold stimulation and air blast tests.

Cold sensitivity test					
Time intervals	Groups	Laser	Gluma	Laser + Gluma	Control
2 W	Laser		0.017		0.002
	Gluma				0.497
	Laser + Gluma	0.055	0.547		0.267
	Control				
1 M	Laser		0.019		0.004
	Gluma				0.686
	Laser + Gluma	0.016	0.652		0.436
	Control				
Air blast test					
1 M	Laser		0.034		0.003
	Gluma				0.040
	Laser + Gluma	0.673	0.771		0.017
	Control

## Discussion

Based on the results of the present study, the null hypothesis was rejected. The average VAS scores for cold stimuli were not significantly different between the 4 groups 15 minutes and 1 week after the intervention. This finding concurs with the results reported by Newman et al.^34^ regarding the late effects of topical desensitising substances, including the Gluma Desensitizer. In a clinical trial conducted by Ozlem et al.,^35^ the efficacy of a glutaraldehyde-containing agent was compared with that of Er, Cr:YSGG, and Nd:YAG lasers, as well as their combination, in the treatment of CDH. The results showed no significant difference between the groups 30 minutes after the intervention.

Nonetheless, in our study 2 weeks and 1 month after the intervention; the laser group showed the most significant reduction in the VAS score. Moreover, a study by Vazirzadeh et al.^36^ compared the efficacy of 5% sodium fluoride, 940-nm diode laser, and Gluma in blocking dentin tubules. They found that 940-nm diode laser had a greater effect on sealing the dentin tubules compared to the other 2 treatments, that may be attributed to the washing away of topical substances (eg, Gluma Desensitizer) over time. Similarly, a study by Khoubrouypak et al.^14^ indicated that laser alone was more effective in occluding open dentin tubules compared to the combined application of laser and Gluma. However, it should be noted that they used 810-nm diode laser, which is different from our 980-nm diode laser. Overall, their results revealed that Gluma prevents further penetration of diode laser into dentin tubules and diminishes its effects compared to laser irradiation alone. In a clinical study by Hashim et al.^37^, they used an 810-nm diode laser with irradiation durations of 30 and 60 seconds for the treatment of CDH. They concluded that pain completely disappeared after 1 week of irradiation, which contradicts our results and may be related to differences in diode laser wavelengths.

Based on our results, 3 months after the intervention, the average VAS score for cold stimuli was not significantly different between the 4 groups. A study by Ehler et al.^38^ supported our findings, indicating no significant difference between the Gluma and Er:YAG laser groups after 3 or 6 months of application. Additionally, the results from a meta-analysis conducted in 2020, indicating non-significant change in diode laser versus topical desensitising agents after 3 months of the application.^22^

In our study, the similarity in the decline process of average VAS score between the intervention groups after 3 months may be due to the shared mechanisms of high-power laser and Gluma, both of which function by closing the dentin tubules. It can be also suggested that the rate at which the Gluma dissolves in the oral environment is similar to the effect of laser, resulting in no significant differences between the groups 3 months after the intervention. Also, in the present study, the decline process of average VAS scores for cold and air stimuli were similar. However, differences in the results between these 2 stimuli may be due to their differing mechanisms of stimulation, as cold stimuli cause the outward movement of dentin fluid and typically elicit more intense responses than air stimuli, which cause the inward movement of dentin fluid.^39^

It is important to consider that differences in the study design, including clinical and non-clinical implementation methods, study duration, the type and power of lasers used, and the used sensitivity tests, can hinder the accurate comparison of results across different studies.^40^ It should be noted that the response to pain is mostly subjective and differs from 1 person to another, depending on the individual's pain threshold, which may have a prominent effect on the randomised clinical trial results. Also, assessment of CDH is not a straightforward task, as the exposed dentin tubules are not always sensitive, and natural desensitisation may occur over time due to obstruction by dental calculus and salivary proteins.^41^ The restricted sample size in our study stemmed primarily from patients' reluctance to participate, posing a limitation in our research. Moreover, factors such as oral hygiene, tooth brushing habits, excessive acidic diet contribute to the improvement of CDH which cannot be fully controlled solely through patient advising and instructing throughout the 3 months of study. The 980-nm laser demonstrated greater efficacy in treating dentin sensitivity compared to both Gluma and the combined approach. Nonetheless, it is crucial to emphasise the safety profile, particularly ensuring the safety of the pulp during laser irradiation.^42^

Nevertheless, due to the affordability and ease of use of topical desensitising agents like Gluma, clinicians are recommended to use them as a routine treatment for CDH and, lasers can be used in cases where topical desensitising agents are ineffective.^43^ We suggest that future studies explore the use of high-power diode lasers in combination with other desensitising agents, such as carbonate and fluoride compounds, in a larger sample size with a longer follow-up.

## Conclusions

Based on the results of the present study, both high-power 980-nm diode laser and Gluma Desensitizer, used alone or in combination, were effective in reducing sensitivity to cold stimuli in CDH patients. The 980-nm diode laser alone was the most effective method, while the Gluma Desensitizer group showed the highest sensitivity to cold stimuli 1 month after the intervention. Therefore, diode lasers can effectively reduce CDH-related pain in patients. If diode lasers are not available, the Gluma Desensitizer can be an effective alternative. However, our results were not consistent over a 3-month period, and further research is needed to determine their stability more definitively.

## Declaration of competing interest

The authors declare that they have no conflict of interests.
